# The Integration of Deep Learning in Radiotherapy: Exploring Challenges, Opportunities, and Future Directions through an Umbrella Review

**DOI:** 10.3390/diagnostics14090939

**Published:** 2024-04-30

**Authors:** Andrea Lastrucci, Yannick Wandael, Renzo Ricci, Giovanni Maccioni, Daniele Giansanti

**Affiliations:** 1 Department of Allied Health Professions, Azienda Ospedaliero-Universitaria Careggi, 50134 Florence, Italy; andrea.lastrucci@unifi.it (A.L.); wandaely@aou-careggi.toscana.it (Y.W.); riccire@aou-careggi.toscana.it (R.R.); 2 Centre TISP, Istituto Superiore di Sanità, 00161 Rome, Italy; gvnnmaccioni@gmail.com

**Keywords:** deep learning, radiotherapy, digital radiology, artificial intelligence

## Abstract

This study investigates, through a narrative review, the transformative impact of deep learning (DL) in the field of radiotherapy, particularly in light of the accelerated developments prompted by the COVID-19 pandemic. The proposed approach was based on an umbrella review following a standard narrative checklist and a qualification process. The selection process identified 19 systematic review studies. Through an analysis of current research, the study highlights the revolutionary potential of DL algorithms in optimizing treatment planning, image analysis, and patient outcome prediction in radiotherapy. It underscores the necessity of further exploration into specific research areas to unlock the full capabilities of DL technology. Moreover, the study emphasizes the intricate interplay between digital radiology and radiotherapy, revealing how advancements in one field can significantly influence the other. This interdependence is crucial for addressing complex challenges and advancing the integration of cutting-edge technologies into clinical practice. Collaborative efforts among researchers, clinicians, and regulatory bodies are deemed essential to effectively navigate the evolving landscape of DL in radiotherapy. By fostering interdisciplinary collaborations and conducting thorough investigations, stakeholders can fully leverage the transformative power of DL to enhance patient care and refine therapeutic strategies. Ultimately, this promises to usher in a new era of personalized and optimized radiotherapy treatment for improved patient outcomes.

## 1. Introduction

### 1.1. Background

Radiotherapy is a clinical sector based on the therapeutic use of ionizing radiation. It represents one of the most important methods of anti-tumor therapy in the various oncological sectors [1,2]. Among the sectors in which it is most used, we find the sector of gynecological tumors, of the rectum and anus, of the lungs, and of the head and neck area. However, the new equipment allows this technological method to also be used in many other sectors, such as, to name a few: sarcomas, primary and secondary tumors of the brain and spinal cord, breast cancer, pancreatic tumors, primary and secondary liver tumors, and other categories. This therapy can be used both exclusively and in combination with other therapeutic methods.

The technological approach is based on a range of solutions that offer different opportunities, and the most recent results have made it possible to develop different radiotherapy modalities with the objective of increasing focus on ensuring better control of the disease and minimizing the medium- and long-term adverse effects. All this requires an increasingly continuous investment in high technological specialization and new experimentation.

Among the dominant methods, we find, for example [3,4,5,6,7]:

1. *Traditional radiotherapy*
  The patient undergoes radiotherapy every day for five days a week, and for a number of weeks that varies based on the disease and parameters, which depend on the tumor to be treated [3].
2. *Conformal radiotherapy*
  It differs from the previous one due to the use of a linear accelerator equipped with a multi-leaf collimator, which allows for better focusing on the neoplasm to be treated while minimizing damage to the surrounding healthy tissues [4].
3. *Intensity-modulated beam radiotherapy (IMRT)*
  It has the use of a multi-leaf collimator in common with conformal radiotherapy. In this case, however, the collimator blades move over the area to be irradiated during irradiation with a pre-established sequence monitored by the computer, while the machine delivers the radiation beam. This allows for greater precision than conformal radiotherapy [5].
4. *Stereotactic radiotherapy*
  It is based on greater attention and accuracy to the immobilization of the patient [6]. It is indicated for very particular clinical cases and a very high specialization of the centers where it is used. In this case, particular accelerators are used, which use a particular device called a gamma knife (gamma ray scalpel).
5. *Hadrontherapy*
  This technology uses subatomic particles, such as protons and ions, capable of irradiating the disease with extreme precision and with different biological effectiveness [6]. In this case, as in the previous one, the therapeutic indications are diverted toward very selected cases [7].

These different methods are now the subject of numerous 360-degree comparative studies by the scientific community [8,9], with a particular focus on innovative dose minimization approaches [10].

### 1.2. Introduction and Potential Applications of Deep Learning in Radiation Therapy

Regardless of which of the five technological methods will be used, radiotherapy is preceded by complex planning. For example, important parameters come into play, such as [11]:

- The PTV (planning target volume), which represents the target volume associated with the body region to be irradiated.
- The GTV (gross tumor volume), which is associated with the actual tumor mass and is related to the PTV.
- The CTV (clinical target volume), which includes the neighboring regions with healthy tissue. This parameter is also related to PTV.

PTV has a central role in radiotherapy, and for some time we have been trying to support its selection with algorithms and/or image processing software [11]. The development of deep learning (DL) has brought new life to all this.

Deep learning, through the use of neural networks with multiple interconnected layers, allows the processing of an enormous amount of data and learning from them [12,13].

Software solutions for contouring based on DL and automated segmentation of anatomical structures are becoming increasingly widespread and seem to offer clear advantages in clinical application [14]. The use of DL-based auto-contouring tools can save a significant amount of time in the procedure, minimize manual corrections, represent a third-party solution for standardizing procedures, and minimize collateral exposure of healthy tissue. In recent years, we have witnessed important developments in this area also driven by the boom in interest that has spread toward the use of artificial intelligence in the health domain sector with the explosion of the COVID-19 pandemic [15,16].

### 1.3. The Rationale and the Purpose of the Study

Some important questions are resonating around the introduction of AI and, in particular, DL in this area, such as:

- *What is the state of development and diffusion of DL in radiotherapy?*
- *What are the opportunities and the obstacles encountered and the challenges to overcome them?*

Systematic reviews are an important basis for making a map point since they are an index of the consolidation of emerging themes and an indirect index of the themes that require greater concentration of scholars.

The objective of this study is, therefore, to carry out, through a narrative review, an umbrella review [17,18], which allows an analysis of the systematic reviews produced to date in this area to answer these questions.

Performing an umbrella review based on a narrative review of systematic reviews is vital for consolidating evidence and identifying both emerging themes and patterns. It enables a nuanced understanding of the research landscape, an exploration of heterogeneity, and the identification of gaps [17,18].

### 1.4. Organization of the Study

The study is structured into five sections, including the introduction in Section 1.

Section 2 outlines the umbrella review design, illustrating the methodology employed to identify each systematic review included in the study.

Following this is Section 3, presenting the results, which are divided into subsections.

Section 3.1 conducts an analysis of trends in scientific production in this field over time.

Section 3.2 provides a detailed analysis, categorizing each study from the overview, supported by tables (Table 1 and Table 2).

Additionally, Section 3.2 includes an excerpt from each analyzed study.

Section 4 is devoted to the discussion, organized into seven subsections. Section 4.1 presents preliminary insights, offering initial observations and understandings.

Section 4.2 critically explores emerging opportunities, examining potential paths for further investigation or development.

In Section 4.3, specific areas warranting deeper examination are identified and discussed.

Section 4.4 elaborates on key areas where a collective need for more in-depth exploration is recognized. This subsection is further subdivided into detailed paragraphs, providing targeted analyses derived from the literature.

Section 4.5 introduces and offers commentary on a comprehensive synoptic diagram, conclusively summarizing the umbrella review project. Following this, the takeaway message (Section 4.6) and limitations (Section 4.7) of the study are presented.

Finally, Section 5 is dedicated to the conclusions.

## 2. Methods

This umbrella review, based on a narrative review, used the ANDJ standardized checklist designed for narrative reviews [19]. Such a narrative checklist is a methodological tool that provides detailed and structured guidance during the review process. It aids in standardizing the review process by establishing key criteria for use during the analysis, making the process of constructing the study transparent.

The PubMed and Scopus databases were used in the overview. A qualification methodology was used to choose the studies based on an assessment of qualified parameters [20]. Based on [20], we evaluated each contribution based on six key parameters:

1. Clarity of study rationale in the introduction,
2. Appropriateness of the work’s design,
3. Clarity in describing methods,
4. Clear presentation of results,
5. Justification and alignment of conclusions with results,
6. Adequate disclosure of conflicts of interest by authors,
7. The scoring system involves assigning graded scores (1 = min; 5 = max) to each one of the first five parameters based on the quality of each criterion.

For the last parameter, a binary assessment (Yes/No) was conducted regarding the disclosure of conflicts.

To preselect studies:

- Each of the first five parameters must obtain a minimum score of 3,
- The last parameter must be marked “Yes” for conflict disclosure.

Only peer-reviewed studies were considered.

We used the keys “radiotherapy” and “deep learning” combined in AND logic and with searches both in the title/abstract and the full text.

We identified 18 studies [21,22,23,24,25,26,27,28,29,30,31,32,33,34,35,36,37,38].

## 3. Results

Below is an analysis of the trends of the studies in this field, reported in Section 3.1, a detailed analysis, with a categorization of the overview of each study supported by tables (Table 1 and Table 2), in Section 3.2, and a summary in Section 3.3.

### 3.1. The Trends in the Studies on Deep Learning in Radiotherapy

**Figure 1:**
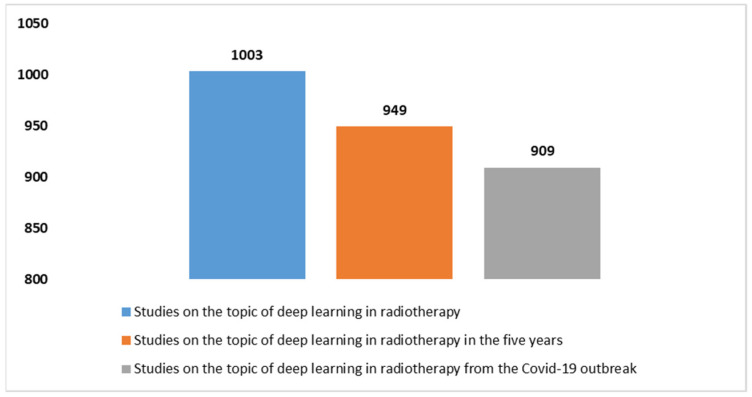
Studies focusing on deep learning and radiotherapy.

**Figure 2:**
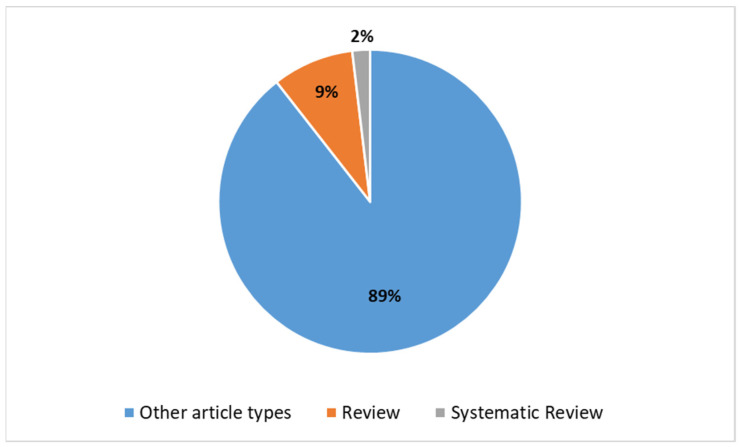
Article types focusing on deep learning and radiotherapy.

A search was conducted using specific criteria (see Box 1) on the PubMed database, resulting in a total of 1003 studies on the use of DL in radiotherapy from 2013 to the present. Figure 1 shows the increasing number of articles found in PubMed related to the application of DL in radiotherapy, based on the selected search parameters. Figure 2 presents the distribution of article types, with reviews (*n* = 87) being more prevalent than systematic reviews (*n* = 19), in relation to DL in radiotherapy.

Box 1 **The proposed composite keys.**

           *(radiotherapy[Title/Abstract]) AND (deep learning[Title/Abstract])*

                  *(radiotherapy[Title/Abstract])*

Research in this field has significantly accelerated during two distinct periods, as depicted in Figure 1. The first surge occurred in the past five years (from 2019 to now), with 94.6% of all indexed articles on this topic being published on PubMed. This period highlighted the growing interest and collaborative efforts to enhance knowledge and understanding of DL in radiotherapy.

The onset of the COVID-19 pandemic marked an even faster growth phase, with nearly all published articles (90.6%) since 2020 focusing on this topic.

The COVID-19 pandemic has caused a surge in the use of DL in the medical field, particularly in radiotherapy. This increase in research activity highlights the ability of the academic and research communities to respond quickly and effectively to the challenges of the pandemic. It also showcases their ability to drive innovation during times of crisis.

**Figure 3:**
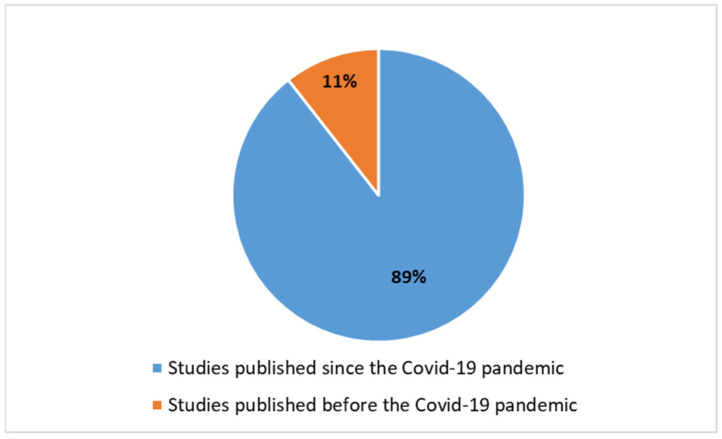
Number of articles published about the use of DL in RT pre- and post-COVID-19 pandemic.

The rise in publications on DL in radiotherapy on PubMed is a result of advancements in technology and a growing recognition of DL’s potential to improve the radiotherapy workflow. By streamlining processes, DL is at the forefront of transforming radiotherapy practices, making them more personalized, accurate, and efficient. The emphasis placed by researchers on this subject has been markedly intense from 2020 to the present, as demonstrated in Figure 3. This figure indeed reveals that interest among researchers has significantly surged since the COVID-19 pandemic, with approximately 90% of articles concerning the application of DL in radiotherapy being published since the onset of the COVID-19 pandemic (*n =* 897).

In conclusion, the growing body of literature on DL in radiotherapy, especially in light of the COVID-19 pandemic, emphasizes the important role of DL in shaping the radiotherapy workflow. This trend not only showcases the field’s ability to adapt and innovate in response to emerging challenges, but also signifies the increasing acknowledgment of DL as a crucial element in continuously improving and refining radiotherapy techniques.

### 3.2. Outcome from the Analysis

#### 3.2.1. General Findings from the Analysis

The collective message that emerged from the analysis of the systematic reviews underscores a profound development in the medical field, particularly within the domain of radiotherapy, propelled by the advent of cutting-edge technologies, such as artificial intelligence (AI), deep learning (DL), and convolutional neural networks (CNNs) [38]. These systematic reviews meticulously delineate the multifaceted impact of these technologies on various aspects of healthcare, encompassing clinical practice, the education of healthcare professionals, and the broader healthcare landscape [25].

A salient theme elucidated throughout these reviews is the immense potential of DL to catalyze a paradigm shift in radiotherapy workflows. DL, especially in its manifestation within the field of AI, emerges as a pivotal player, offering substantial enhancements across diverse stages of radiotherapy—ranging from pre-treatment planning to real-time treatment delivery and post-treatment evaluation [38]. Through a comprehensive analysis of the systematic reviews, it becomes evident that DL’s scope within radiotherapy spans a myriad of topics. Notably, DL methodologies empower significant advancements in predicting treatment outcomes [38], streamlining and automating contour delineation [21,33,35,37], and optimizing treatment planning procedures [21,24,32,35,37].

Moreover, the integration of DL into radiomic analyses represents a promising avenue for clinicians, enabling the extraction of intricate features from medical imaging data. This integration is thoroughly evaluated in several systematic reviews [22,26,27,36,38], which underscore its potential to enhance diagnostic precision and prognostic assessments. Furthermore, these reviews illuminate that the development of radiomics not only aims to augment the accuracy of diagnostic and prognostic evaluations but also endeavors to tailor radiotherapy interventions to the unique characteristics of individual tumors, potentially leading to superior clinical outcomes and mitigated side effects.

The imperative of standardizing DL integration looms large in the specialist literature, alongside considerations regarding user-friendliness and technical nuances [22,26,34]. These systematic reviews underscore the critical necessity of establishing clear guidelines for the seamless integration of DL into existing systems, thereby addressing challenges pertaining to user interface, user experience, and the technical intricacies associated with the deployment of AI technologies in healthcare.

Each systematic review, including those conducted by Boldrini et al. [38] and Huang et al. [25], accentuates the exigency for further research to comprehensively grasp the clinical implications and intricacies surrounding the integration of DL into routine clinical practice. These studies advocate for intensified efforts to discern how DL can be effectively assimilated into standard daily protocols. Additionally, several systematic reviews underscore the paramount importance of publicly available, high-quality databases and rigorous validation processes to bolster the development of DL across diverse radiotherapy applications [23,26,28,29,32,33,34]. Notably, the review by Booth et al. [32] draws attention to the prevailing challenges of small sample sizes and a high risk of bias in current research endeavors, thereby emphasizing the imperatives for enhanced study quality and refined research methodologies.

In essence, these systematic reviews collectively paint a compelling narrative of a transformative shift in the medical landscape, precipitated by the seamless integration of AI and DL into clinical practice within radiotherapy workflows. A pivotal determinant for augmenting the efficacy of DL in this context lies in the availability of expansive, shared datasets. Such repositories of high-quality data serve as indispensable assets in optimizing DL’s performance and fostering the development of more precise, reliable applications in the realm of radiotherapy [29,33,34].

Table 1 reports the key findings from the analysis of the overview of systematic reviews.

#### 3.2.2. Emerging Categorization from the Analysis

Delving into greater detail, we identified the following areas of interest and/or emphasis, with a focus on the categorization, as reported in Table 2.

These emerging themes/patterns collectively indicate a trend toward the integration of DL to enhance and improve radiotherapy workflow. The systematic reviews also highlight the need for further research, standardization, and overcoming challenges for broader adoption of this technology.

### 3.3. In-Depth Analysis of the Detected Studies: A Comprehensive Overview

To complement our overview, after having identified the themes and focus elements of the studies, here, we report a more far-reaching summary of each individual systematic review.

Almeida et al. [21] highlighted the potential of DL in improving treatment planning for prostate cancer, focusing on automating the contouring process to enhance speed and consistency while maintaining quality. They examined various network architectures based on computed tomography (CT) or magnetic resonance imaging (MRI), noting the field’s rapid growth but also the limitations due to small patient datasets. Despite promising results, there remains a significant gap before these technologies can be fully integrated into clinical practice.

The systematic review and meta-analysis proposed by Kothari et al. [22] assessed the prognostic value of radiomics-based models in non-small-cell lung cancer (NSCLC) treated with radical radiotherapy, finding modest capabilities with a Harrell’s Concordance Index (C-index) of 0.57. They highlighted significant heterogeneity in feature selection and model development across studies and suggested that future research focus on standardized radiomics features, robust methodology, and DL to enhance model performance.

Chlap et al. [23] examined data augmentation techniques for DL models in radiology and radiotherapy, highlighting their necessity due to the reliance on large datasets for training. They categorized augmentation methods applied to CT and MRI medical images into basic, deformable, and DL-based techniques, aiming to enhance model performance and provide insights into their clinical validity. The study underscores the importance of data augmentation in improving DL algorithms where large datasets are scarce.

Spadea et al. [24] evaluated DL-based synthetic CT (sCT) generation methods, grouped into three main clinical applications: (i) replacing CT in MR-based treatment planning, (ii) aiding cone-beam CT in image-guided adaptive radiotherapy, and (iii) creating attenuation maps for positron emission tomography (PET) correction. They covered research from January 2014 to December 2020, detailing each category’s contributions, challenges, and achievements, and assessed the clinical readiness and potential future trends of DL-based sCT methods.

Huang et al. [25] highlighted that the widespread use of computers and data explosion has significantly fueled artificial intelligence (AI) development, with DL algorithms, such as convolutional neural networks (CNNs), offering radiation oncologists promising tools to streamline radiotherapy and reduce their workload. This facilitates more time for advanced decision-making. As DL evolves closer to clinical application, it is crucial for oncologists to understand its principles for effective use. This paper delved into AI’s development, basic concepts, and its potential in radiation oncology, suggesting further growth prospects for DL in this field.

Walls et al. [26] explored the utility of radiomics in guiding clinical decisions for lung cancer radical radiotherapy, highlighting the lack of validated biomarkers for personalized treatment in the face of the disease’s significant mortality. Their systematic review included and analyzed 44 studies that established a connection between radiomic indicators, notably texture features and kurtosis, and clinical outcomes, including disease management, patient survival, and treatment-related toxicity. Despite these insights, obstacles, such as the need for standardized data, improved reporting, and external validation through prospective studies, pose barriers to the practical application of radiomics, emphasizing the critical need for enhanced evaluative frameworks and research methodologies in the field.

Kim et al. [27] conducted a systematic review and meta-analysis to tackle the diagnostic challenge of distinguishing true progression from non-progression in brain metastasis after stereotactic radiotherapy or surgery using MRI. This analysis, encompassing 7 studies with a total of 485 patients, found that AI-assisted MRI, through radiomics, achieved sensitivity and specificity rates of 77% and 74%, respectively. While the overall quality of the studies was deemed favorable, the current diagnostic capabilities of AI-assisted MRI fall short of the reliability needed for clinical use, underscoring the necessity for further research utilizing more sophisticated methodologies and expanded datasets.

Avanzo et al. [28] explored the landscape of AI applications in imaging research in Italy, covering the period of 2015–2020. They highlighted MRI as the most prevalent imaging modality, with a significant focus on neurological diseases and cancer diagnosis. The review noted a dramatic increase in AI research, particularly in classification and segmentation tasks, with a mix of machine learning and DL approaches. The findings underscore the burgeoning interest and need for collaborative frameworks, shared databases, and research guidelines in the AI imaging domain within Italy.

Yang et al. [29] proposed a systematic review and meta-analysis on DL models for cervical cancer CT image segmentation, reviewing 1893 articles and including 14 in the analysis. The study included 14 articles and revealed high accuracy in segmenting clinical target volumes and organs-at-risk, with dice similarity coefficient (DSC) scores ranging from 0.83 to 0.92. Despite good performance and efficiency (segmentation times between 15 s and 2 min), the findings highlighted the need for public, high-quality databases and further large-scale validation for future radiotherapy applications.

Rusanov et al. [30] explored the application of DL in enhancing cone-beam CT (CBCT) image quality for online adaptive radiation therapy (ART), addressing how updated patient anatomy can optimize treatment parameters despite CBCT’s traditional quality limitations. This comprehensive review, covering January 2018 to April 2022, evaluated DL strategies for CBCT correction and synthetic CT generation, emphasizing study designs, DL techniques, image quality, and dosimetric accuracy. They concluded with recommendations for clinicians and DL practitioners, identifying literature gaps and advocating for the integration of state-of-the-art DL methods in radiation oncology.

Booth et al. [31] evaluated the accuracy and quality of machine learning (ML) models, specifically DL, in monitoring biomarkers to assess treatment response in glioblastoma, based on articles from September 2018 to January 2021. Despite the promising diagnostic performance of ML models in differentiating between tumor progression and mimics in glioblastoma using MRI features, the studies suffered from small sample sizes, high bias risk, and applicability concerns, highlighting the need for improved study quality and design. The meta-analysis of ten studies showed moderate sensitivity, specificity, and balanced accuracy, indicating potential yet calling for refinement in research methodologies.

Hasan et al. [32] explored the application of CNNs in ENT radiology, a field where their use is less common compared to other radiology disciplines, possibly due to unfamiliarity within the otolaryngology community. Through a systematic review of thirty articles up to October 2020, they demonstrated CNNs’ high accuracy in tasks such as identifying structures, detecting pathology, and segmenting tumors for radiotherapy in various ENT subspecialties. This study underscores the potential of CNNs to significantly impact clinical practice in ENT radiology by automating and enhancing diagnostic and treatment planning processes.

The systematic review and meta-analysis proposed by Liu et al. [33] investigated the effectiveness of DL algorithms for contouring organs-at-risk in head and neck cancer radiation treatment planning, assessing 22 studies from a pool of 149. The analysis revealed high dice similarity coefficient (DSC) scores across various organs-at-risk, indicating DL algorithms’ accuracy in automating the contouring process, thus potentially reducing the workload for oncologists and enabling more precise radiotherapy plans. The study highlighted the necessity for high-quality datasets and further algorithmic improvements to optimize DL’s performance in clinical settings.

Tan et al. [34] evaluated DL models’ effectiveness in predicting radiotherapy-induced toxicity, analyzing fourteen studies across various cancer types (prostate (*n* = 2), head and neck cancer (HNC; *n* = 4), liver (*n* = 2), lung (*n* = 4), cervical (*n* = 1), and esophagus (*n* = 1)). The review highlighted the utilization of advanced techniques, such as ensemble learning, data augmentation, and transfer learning, in model development. The authors concluded that while DL models show promise in toxicity prediction, future research needs larger, more diverse datasets and standardized methodologies to enhance research consistency and outcomes.

Franzese et al. [35] investigated the role of DL in the radiotherapy workflow for HNC, analyzing 62 selected articles from 2016 to 2022 that covered the entire RT workflow: contouring, planning, and delivery. This systematic review highlighted the significant focus on organ-at-risk segmentation and the need for studies to assess the clinical impact of AI and provide confidence levels for AI predictions. The conclusion underscored AI’s potential in automating the complex radiotherapy workflow for HNC and called for interdisciplinary research to align AI development with clinical requirements.

Eidex et al. [36] analyzed 197 studies up to 31 December 2022, on MRI-guided radiation therapy and DL, categorizing them into image segmentation, synthesis, radiomics, and real-time MRI to fully support ART. This systematic review discussed the clinical significance and challenges of DL in enhancing tumor segmentation, deriving X-ray attenuation from MRI, and improving tumor characterization and motion tracking, emphasizing the rise of multi-modal, visual transformer, and diffusion models in recent trends.

Chen et al. [37] investigated the use of unpaired image-to-image (I2I) translation in medical imaging. Out of 461 studies, 55 were included, showcasing I2I’s role in segmentation, unpaired domain adaptation, denoising, and clinical applications, such as automatic contouring for MRI, CT, and radiotherapy planning. Despite its potential, the limited external validation and the scarcity of publicly available pre-trained models restrict the immediate practical application of these methods in clinical practice.

Boldrini et al. [38] investigated the influence of AI, DL, and radiomics on image-guided radiation therapy (IGRT) in radiation treatments, highlighting their promising roles in diagnosis, treatment optimization, and outcome prediction. Through a systematic search of electronic databases, 84 papers were analyzed, revealing significant contributions of AI and radiomics to IGRT, across 23 and 61 papers, respectively. Despite the reliance on retrospective data, AI and radiomics were shown to significantly enhance IGRT across all radiotherapy workflow phases, indicating a need for further research to solidify their clinical impact and integration into standard treatment protocols.

## 4. Discussion

The section is structured into multiple paragraphs. Section 4.1 presents early insights, providing initial observations and understandings. Section 4.2 delves into the opportunities that have arisen, exploring potential avenues for further investigation or development. In Section 4.3, specific areas warranting deeper examination are identified and discussed. Section 4.4 elaborates on key areas where a collective need for more in-depth exploration is perceived. This section is subdivided into detailed subsections, offering targeted analyses derived from the literature. Section 4.5 introduces and provides commentary on a comprehensive synoptic diagram, conclusively summarizing the umbrella review project. Following this, the takeaway message (Section 4.6) and limitations (Section 4.7) of the study are presented.

### 4.1. Early Insights and Discussion

Radiation therapy has a history dating back to the early last century. The initial studies in this field date back to 1903, and since then, 226,830 studies have been published on PubMed (search key in Box 1, position 2). Over the years, with the advancement of technological innovations, various techniques, such as traditional radiotherapy, conformal radiotherapy, IMRT, stereotactic radiotherapy, and hadrontherapy, have been gradually established, and researchers are still exploring new methods [3,4,5,6,7].

**Figure 4:**
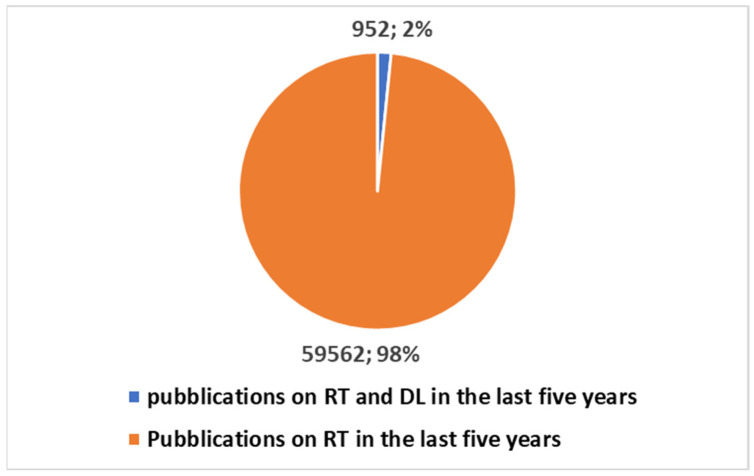
Articles on RT and DL versus articles on RT in the last five years.

Only a small fraction, approximately 0.44%, of these studies (equivalent to 1003, not perceptible in a potential graph) have addressed the introduction of DL, and this trend has only emerged since 2013. The significant push for research in the application of DL in radiation therapy was fueled by the COVID-19 pandemic (Figure 1). From the onset of the pandemic until now, 909 studies have been produced, representing over 90% of the total. Considering the last five years of scientific production, marked almost entirely by the COVID-19 pandemic, the ratio of total scientific production in radiation therapy, considering and not considering DL, has increased from 0.44% to 1.6%, quadrupling the interest and demonstrating a growing focus of researchers on these issues. This trend is now visually perceptible (Figure 4).

**Figure 5:**
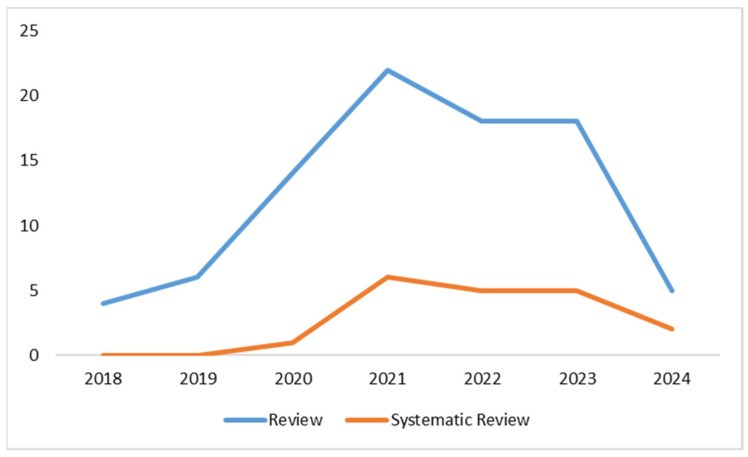
Temporal trends of reviews and systematic reviews published on the PubMed database focusing on deep learning and radiotherapy.

The trend of reviews and systematic reviews, as shown in Figure 5, emphasizes developments in research and the consolidation of specific themes over the past five years through Cartesian graphs. Indeed, these two types of studies serve as indicators of both researchers’ interest and the solidification around topics of interest, reflecting the establishment of medical knowledge. The trends of both types of studies follow a similar pattern, with systematic reviews naturally exhibiting a slight delay compared to the initial studies, as expected. This synchronization in trends implies that as primary studies emerge, systematic reviews follow, providing a comprehensive and reflective overview of the evolving research landscape. The alignment of these trends signifies the ongoing interest of researchers and the firm establishment of knowledge within the medical field.

The accumulation of research trends, evolving themes, and the growing body of knowledge in a particular field serves as a compelling motivation and justification for our umbrella review study. An umbrella review [17,18] involves a comprehensive examination and synthesis of findings from multiple systematic reviews on a specific subject. In this context, our decision to conduct an umbrella review was specifically centered on systematic reviews. By delving into these studies, we aimed to offer a comprehensive overview of the existing body of research, focusing on the highest editorial scientific tools—the systematic reviews—recognized for their thorough analyses, which systematically gather and evaluate evidence, contributing to a comprehensive understanding of the subject matter. The overview revealed opportunities and specific areas requiring in-depth investigations, and perceived global areas needing further research and attention.

### 4.2. Opportunities Explored: Critical Reflections on DL in Radiotherapy

The intersection of deep learning (DL) and radiotherapy presents a dynamic yet critically transformative force, unveiling a plethora of opportunities across multifaceted dimensions of cancer care. Recent investigations [21,22,23,24,25,26,27,28,29,30,31,32,33,34,35,36,37,38] shed light on the expansive vistas that DL illuminates.

DL stands poised to revolutionize the landscape of radiotherapy, offering a myriad of transformative prospects across diverse applications. From automating intricate procedures to refining prognostic models and enhancing imaging methodologies, DL emerges as a disruptive force for innovation. Noteworthy advancements include its application in prostate cancer treatment planning, where DL streamlines the contouring process, promising efficiency and consistency [21]. This not only accelerates processes but also maintains high-quality standards, suggesting a paradigm shift in precision medicine for prostate cancer patients. Similarly, in non-small-cell lung cancer (NSCLC), DL-driven radiomics models enhance prognostic accuracy in radical radiotherapy [22], demonstrating the potential to optimize treatment strategies and outcomes, thus emphasizing DL’s pivotal role in personalized cancer care.

Augmentation techniques for DL models in radiology and radiotherapy address the perennial challenge of limited large datasets, potentially improving performance [23]. DL-based synthetic CT generation methods pave the way for MR-based treatment planning, adaptive radiotherapy, and PET correction [24,25], offering precision and efficiency while optimizing treatment parameters based on updated patient anatomy.

Radiomics assumes a central role in guiding clinical decisions for lung cancer radical radiotherapy, leveraging advanced imaging features for personalized interventions [26]. The integration of radiomic indicators signifies a shift toward more targeted and effective lung cancer treatments. Moreover, in brain metastasis diagnosis, AI-assisted MRI shows promise for enhancing diagnostic capabilities [27], albeit requiring further refinement for clinical deployment.

Italy has witnessed a surge in AI applications in imaging research, particularly in classification and segmentation tasks [28], highlighting the necessity of collaborative frameworks and shared databases to harness the full potential of AI in advancing Italian imaging research.

DL models exhibit promising accuracy in segmenting clinical target volumes for cervical cancer CT images, offering precision in treatment planning [29]. Improving cone-beam CT (CBCT) image quality for online adaptive radiation therapy emerges as a critical opportunity [30], necessitating the integration of state-of-the-art DL methods for optimized treatment.

Monitoring biomarkers through DL in glioblastoma treatment response assessment holds promise for personalized therapeutic strategies [31], potentially reshaping treatment evaluation paradigms.

In ENT radiology, convolutional neural networks (CNNs) emerge as high-accuracy tools for automating diagnostic and treatment planning processes [32]. Similarly, DL algorithms show potential in automating the contouring process for head and neck cancer radiation treatment planning [33], thereby refining precision and reducing oncologists’ workload, leading to enhanced treatment efficiency.

DL models also demonstrate promise in predicting radiotherapy-induced toxicity across various cancer types [34], offering avenues for refining toxicity prediction models and tailoring personalized treatment plans. Moreover, DL’s potential in automating complex radiotherapy workflows for head and neck cancer (HNC) presents transformative opportunities [35], with applications in organ-at-risk segmentation and workflow automation for enhanced HNC care.

DL in MRI-guided radiation therapy holds potential for improving tumor segmentation, deriving X-ray attenuation from MRI, and enhancing tumor characterization and motion tracking [36], promising precision and innovation.

Unpaired image-to-image translation in medical imaging emerges as a potential game-changer [37], with opportunities for exploring novel applications and methodologies in clinical practice, leading to advancements in segmentation, adaptation, and denoising.

The integration of AI, DL, and radiomics significantly influences image-guided radiation therapy (IGRT) across all workflow phases [38], offering prospects for further applications in diagnosis, treatment optimization, and outcome prediction for enhanced IGRT.

In summary, the transformative opportunities brought forth by DL in radiotherapy are extensive and profound. Collaborative endeavors, standardized methodologies, and a dedication to surmounting existing challenges are imperative to fully capitalize on these opportunities. As technology progresses, the potential to improve patient outcomes and streamline oncological care becomes increasingly tangible, awaiting exploration and implementation. In essence, the panorama of opportunities ushered in by DL in radiotherapy is vast and transformative, necessitating critical reflection and concerted efforts to realize its full potential [21,22,23,24,25,26,27,28,29,30,31,32,33,34,35,36,37,38]. Table 3 provides a synthesis of these emerging opportunities.

### 4.3. Limitations Explored: Critical Reflections on DL in Radiotherapy

While the overview presented promising opportunities, it is imperative to confront the specific limitations existing within the field and underscore the areas requiring further scrutiny and refinement. The impact of AI and radiomics on image-guided radiation therapy (IGRT) appears substantial, promising advancements in treatment planning and precision radiotherapy [38]. However, the reliance on retrospective data in the literature casts doubt on the validity of the findings, necessitating rigorous external validation studies to establish their credibility. Confirmation of the potential of these tools and their alignment with clinical outcomes and gold-standard treatment strategies is paramount for their practical deployment. Automated contouring technology driven by DL algorithms emerges as a potent tool for contouring head and neck organs-at-risk (OARs), purportedly reducing the workload of radiation oncologists and facilitating precision radiotherapy [33]. However, the identified need for constructing high-quality datasets highlights the imperative of optimizing datasets to bolster DL algorithm performance. Ongoing research endeavors should prioritize algorithm refinement and innovative approaches to ensure consistent accuracy.

Toxicity prediction employing DL techniques demonstrates consistent performance, suggesting their potential utility in radiotherapy [34]. However, the call for future research with large and diverse datasets and standardized study methodologies underscores the necessity of overcoming challenges to enhance the reliability and consistency of research outputs in this domain. The automation of the radiotherapy workflow for head and neck cancer (HNC) treatment holds transformative potential [35]. Interdisciplinary collaboration, involving both clinicians and computer scientists, is advocated for future studies, ensuring the effective alignment of AI technologies with clinical needs, and fostering a comprehensive understanding of their clinical impact.

In ENT radiology, the deployment of AI methodologies shows promise in various applications, including nodule and tumor identification, anatomical variation identification, and tumor segmentation [32]. This evolving field anticipates further evolution and integration into everyday practice. However, to fulfill its potential, continuous refinement of technologies and methodologies is indispensable. The translation of medical images through image-to-image (I2I) models presents valuable applications for medical physicists [37]. Nevertheless, the dearth of external validation studies and pre-trained models hampers immediate practical application. Initiatives aimed at overcoming these limitations, such as increased external validation and broader availability of pre-trained models, are imperative for advancing these techniques in practice.

Deep learning models demonstrate high accuracy in the automatic segmentation of cervical cancer CT images, offering promise for future radiotherapy applications. Nonetheless, the need for public high-quality databases and large-scale research verification underscores the importance of collaborative efforts to enhance the reliability and applicability of these models.

Future research endeavors should prioritize the development of improved tools for the evaluation of radiomics studies, addressing issues such as standardization of input scan data, quality of reporting, and external validation in randomized clinical trials. Machine learning models utilizing MRI features for distinguishing progression from mimics demonstrate good diagnostic performance, but the need for improvement in study quality and design is evident [31]. Future studies should focus on refining methodologies to strengthen the diagnostic capabilities of AI in MRI-based applications. The evaluation of DL-based synthetic CT (sCT) generation reveals its potential benefits and clinical readiness [24]. Future studies should delve deeper into the clinical applicability of these methods, ensuring they meet the required standards for safe and effective use. As artificial intelligence models trained using augmented data move toward clinical implementation, continuous refinement and validation are essential. The review anticipates the evolving landscape of AI techniques and aims to instill confidence in the validity of the models produced. The diagnostic performance of AI-assisted MRI remains inadequate for reliable use in clinical practice [27]. The identified need for future studies with improved methodologies and larger training sets emphasizes the ongoing efforts required to enhance the reliability and effectiveness of AI-assisted MRI. The surge of interest in AI applied to imaging in Italy highlights the need for collaborative frameworks, shared databases, and research guidelines [28]. These initiatives are crucial to fully harness the potential of AI applications in medical imaging. Radiomics-based models for lung cancer demonstrate modest prognostic capabilities [22]. Future research should focus on standardizing radiomics features, robust feature selection, and incorporating DL techniques to improve the predictive accuracy of imaging-based models. The comprehensive review outlining the development and application of AI in radiation oncology emphasizes the need for ongoing research and clarifies the potential for further DL development in this field. The recognition of this potential paves the way for continued exploration and advancements. Although models have achieved satisfactory results, there is acknowledgment of the need for improvement before safe and effective clinical application. This recognition underscores the commitment to continuous refinement and enhancement in the journey toward practical implementation. Overall, the discourse on DL in radiotherapy reflects a dynamic landscape with transformative potential. While opportunities are abundant, the identified limitations underscore the importance of ongoing research, interdisciplinary collaboration, and standardization efforts. Addressing these challenges will be instrumental in unlocking the full potential of DL in this field, ultimately enhancing patient outcomes and advancing the field of radiotherapy.

Table 4 reports the specific emerging suggestions for a broader investigation.

### 4.4. Navigating Critical Focus Areas: Global Explorations and Expansion of Literature Analysis in Deep Learning Applications in Radiotherapy

From the emerging studies, critical areas that demand further research in this field came to light. These aspects primarily highlight the strong interconnection between radiotherapy and digital radiology, suggesting the need for deeper investigations considering this interdependence.

#### 4.4.1. Navigating Critical Focus Areas in Deep Learning Applications in Radiotherapy

The extensive analysis conducted across a multitude of studies underscores with utmost gravity the critical imperatives that lie at the heart of advancing DL within the realm of radiotherapy [21,22,23,24,25,26,27,28,29,30,31,32,33,34,35,36,37,38]. These pivotal focal points, which include the paramount importance of standardization, the establishment of robust frameworks, unfettered access to diverse and comprehensive datasets, rigorous external validation protocols, and the adherence to methodological standardization, stand as the bedrock upon which the reliability, efficacy, and broader applicability of DL models in this intricate domain hinge.

First and foremost, the resounding call for “standardization” reverberates throughout the corpus of literature, as elucidated in [36]. The glaring absence of standardized protocols governing data collection methodologies and analysis procedures poses a formidable barrier, significantly impeding the validity and practical utility of DL technologies across radiotherapy applications [36]. This unequivocal realization underscores the urgent necessity for a cohesive, uniform approach that ensures coherence and comparability across diverse research endeavors and clinical implementations alike.

Furthermore, the fervent advocacy for collaborative “frameworks and guidelines” echoes resoundingly, particularly in the context of burgeoning interest witnessed in nations such as Italy [28,35]. The burgeoning landscape of AI applications within Italy’s dynamic imaging research sphere underscores the indispensable need for collaborative frameworks, shared repositories of data, and meticulously crafted research guidelines aimed at harnessing the transformative potential of AI within the realm of medical imaging [28]. Similarly, concerted initiatives are urgently called on for the establishment of common frameworks, centralized repositories of data, and collaborative networks within Italy, all aimed at fostering the rapid advancement of AI research [35]. These compelling insights underscore the pivotal role played by collaborative endeavors and meticulously structured frameworks in driving forward the frontiers of DL application within the intricate domain of medical imaging and radiotherapy.

Moreover, the profound significance of unfettered access to “diverse and standardized datasets” emerges as a recurrent, overarching theme, particularly in the context of confronting the formidable challenges posed by limited data availability [23,28]. The acute shortage of expansive, heterogeneous datasets poses a formidable impediment to the widespread adoption of DL methodologies across various applications, including but not limited to prostate cancer treatment planning and radiology, thereby impeding the refinement and robustness of DL models [23]. This pressing need for diverse datasets is further underscored within Italy’s dynamic imaging research landscape, where collaborative frameworks are identified as indispensable vehicles for harnessing the burgeoning interest witnessed in AI applications [28]. These profound insights serve to underscore the indispensable role played by comprehensive datasets in augmenting the efficacy, generalizability, and real-world applicability of DL models across the expansive spectrum of radiotherapy applications.

Equally crucial is the unequivocal recognition of “external validation” as an indispensable linchpin for ensuring the reliability, validity, and immediate applicability of DL models [27]. The dearth of comprehensive external validation studies, particularly in the context of image-to-image models, is acknowledged as a glaring deficiency, underscoring the imperative need for further validation endeavors aimed at bolstering the immediate applicability and real-world efficacy of proposed methodologies [27]. External validation thus emerges as a pivotal step in cementing the credibility, robustness, and generalizability of DL models, thereby instilling much-needed confidence in their real-world performance across diverse radiotherapy applications.

#### 4.4.2. Harmonizing Challenges between Digital Radiology and Radiotherapy

The above considerations align with the broader trends seen in the integration of artificial intelligence (AI) into digital radiology [39,40,41], a phenomenon that can be said to encompass and permeate various sectors, including radiation therapy [41,42,43,44,45,46].

The integration of artificial intelligence (AI) into digital radiology, encompassing radiotherapy as an integral component, has evolved into a prevalent and transformative theme. Recent studies, exemplified by Giansanti and Di Basilio [39], accentuate the challenges, acceptance, and consensus associated with the incorporation of AI in both digital radiology and radiotherapy. Their work not only highlights the technological advancement but also emphasizes a paradigm shift that significantly influences medical imaging practices, presenting crucial challenges and areas that warrant in-depth studies and research.

Moreover, the comprehensive exploration of regulatory aspects pertaining to AI in digital radiology, which is equally applicable to radiotherapy, is evident in Giansanti’s comprehensive review [40]. This review underscores substantial challenges and bottlenecks in the regulatory landscape, emphasizing the need for a thorough understanding of these aspects in both domains.

In the realm of radiation oncology, the impact of digital radiology has been acknowledged since the early 1990s [41]. Aznar et al. [42] provided a contemporary perspective on radiation oncology in the digital era, emphasizing its transformative nature and the escalating reliance on digital technologies within radiation oncology practices. The pivotal role of Digital Imaging and Communications in Medicine—Radiation Therapy (DICOM-RT) in radiology informatics was highlighted by Law and Liu [43]. Their discussion underscores the significance of DICOM-RT in radiation therapy, emphasizing the importance of standardized protocols for managing and sharing medical imaging data. The longstanding integration of digital radiology into radiotherapy, anchored in [44], has been a fundamental aspect of treatment planning and delivery. Van den Berge et al. [44] elaborated on the significance and challenges associated with digital radiology in radiotherapy, tracing its evolution over the years. This interconnection is sustained with the introduction of DL, where its advent significantly influences both digital radiology and radiation therapy [45]. Sahiner et al. [45] reinforced the interplay between digital radiology and radiotherapy, emphasizing the potential for DL to revolutionize image analysis and treatment planning. The utilization of digital radiology in the planning and delivery of radiation therapy remains a subject of continued interest [46]. Kalet and Austin-Seymour [46] delved into the intricate role of digital radiology in radiation therapy, emphasizing the critical importance of accurate imaging data for effective treatment planning and delivery.

#### 4.4.3. Insights into Key Areas: A Comprehensive Exploration

The need for standardization, frameworks and guidelines, access to diverse and standardized datasets, and the importance of external validation have been specifically mentioned in some studies but are a vaguely perceived necessity in all the analyzed studies. All of this is in line with the common sentiment in digital radiology, upon which, as previously seen, DL applied to RT builds its foundation.

##### Focus on Ethics

The issues related to standardization and the need for guidelines and shared frameworks are strongly highlighted, for example, with emphasis on ethics in [40,47,48,49,50].

Mudgal et al. [47] conducted an in-depth study focusing exclusively on ethical considerations surrounding AI deployment. Their review covered ethical issues related to harmless and justifiable AI integration across training, incorporation into the health sector, and regulatory frameworks. Emphasizing the need for centralized and non-dispersive data management, the authors advocated for thorough evaluation, refinement, purification, and representation of all demographics. Transparency and security were highlighted as pivotal, and the authors stressed the importance of following authorization processes set by competent authorities.

In a related vein, Harvey et al. [48] explored the transformative potential of AI in digital radiology, addressing nascent phases and untested aspects within clinical spaces. The study outlined challenges, including the evolving legal–regulatory environment, impacting AI introduction. Issues such as FDA approval pathways, government oversight, privacy concerns, ethical dilemmas, and practical considerations in radiologist practice were discussed. Notably, the study underscored the need for careful considerations in nuclear medicine, encompassing reliability, safety, non-maleficence, beneficence, justice, fairness, data privacy, security, confidentiality, bias minimization, clear communication, autonomy, and clarification ability.

Jaremko et al. [49] contributed a study for the Canadian Association of Radiologists, focusing on both regulatory and ethical facets. Their work presented a comprehensive framework outlining legal and ethical issues, with specific reference to the Canadian health domain.

Currie et al. [50] centered their review specifically on nuclear medicine. They highlighted the substantial opportunities associated with “ethical AI,” emphasizing its potential to enhance productivity, workflow, research capabilities, and clinical applications.

##### Focus on Regulatory Frameworks

The issues related to standardization and the need for guidelines and shared frameworks are strongly emphasized, along with the necessity for standardizing datasets, for instance, with a focus on regulatory frameworks in [39,48,49,51,52,53,54,55].

Two studies focused on regulatory frameworks in both the European Union and the US. Pesapane et al. [53] analyzed medical device (MD) regulations in both regions, noting the European emphasis on cybersecurity, data protection, and MDs’ integration, while the US FDA prioritized data processing, consent, and user/consumer consent. Muehlematter [52] expressed concerns about the approval processes for AI-based MDs in both regions, highlighting an increase in approved MDs but suggesting a lack of a well-defined pathway. Transparency and a dedicated database for MDs were recommended.

Harvey et al. [48] examined the US context, addressing the FDA’s innovative approach to streamline AI approval by adopting a total product lifecycle method. Jaremko et al. [49] provided a study on Canada, discussing regulatory and ethical recommendations for patient data, algorithms, and healthcare practices.

The concept of AI in digital radiology being treated as medical devices (MDs) was emphasized in [52,53]. Arora et al. [54] highlighted AI’s recognition as “Software as a Medical Device (SaMD)” and its interconnection with the Internet of Things, genetic data, and patient records. Allen et al. [55] warned about biases in commercial algorithms affecting gender, ethnicity, and social factors in healthcare. They proposed a solution involving collaboration among institutions to develop robust shared datasets and algorithms with standards to mitigate biases.

##### Focus on Bottlenecks

On these topics, including the need for evaluation of the retrieval of validated external datasets, we found the identification of bottlenecks in [39,40,56,57,58,59]. Three studies on regulatory aspects [56,57,58] provided complementary and comprehensive insights, emphasizing the critical need for external datasets. Alexander et al. [57] examined the impact of workload on decision correctness in radiology, underscoring the importance of regulating workloads with a scientifically sound approach. They cautioned that inadequate regulation could pose more risks than having no regulation at all, especially when considering the influence of AI on decision speediness.

Mezrich [56] delved into legal liability associated with errors linked to AI use. Highlighting critical issues, the review focused on enforcing AI-based product liability laws in the US, uncovering ambiguities that could significantly impact the integration of AI into the health domain and erode trust among stakeholders.

Ebrahimian et al. [58] concentrated on FDA-regulated AI algorithms, reviewing 127 regulated software to classify available information. They observed a growing number of FDA-regulated medical devices (MDs) from 2008 to 2021. Crucially, their review emphasized a lack of sufficient public data on validation/testing datasets for various algorithms, rendering applications in healthcare unjustifiable due to potential generalization and biases. The consensus across these reviews underscores the urgent need for transparent and comprehensive external datasets to ensure the safe and effective integration of AI in healthcare.

##### Focus on Consensus and Acceptance

An alternative viewpoint on these matters is discussed in [39,59,60,61,62,63,64,65,66,67,68,69,70,71,72], emphasizing the significance of consensus-building and acceptance initiatives. These initiatives are not solely targeted at professionals involved in the field but extend to patients, caregivers, and patient associations. The recognition of the importance of involving a broader spectrum of stakeholders underscores a holistic approach to address and incorporate diverse perspectives, fostering a more inclusive and well-rounded understanding of the challenges and advancements in the discussed topics.

Three studies [59,60,61] focused on patient questionnaires, exploring various aspects of artificial intelligence (AI) integration. Lennartz et al. [59] investigated opinions on AI integration throughout the medical workflow, while Zhang et al. [60] conducted interviews on AI in diagnostics, revealing positive considerations with some cybersecurity concerns. Ongega et al. [61] examined perceived perspectives on AI, emphasizing patients’ importance, social factors’ impact, and the utility of questionnaires as sensors.

Hendrix et al. [62] concentrated on primary care providers, highlighting the importance of sensitivity and other parameters in AI-supported clinical reports. AI was deemed suitable for triage roles without radiologist validation.

Other studies [63,64,65,66,67,68] explored the opinions of other stakeholders, including radiography students. Abuzaid et al. [63] found enthusiasm for AI integration in training programs but concerns about job security. Abuzaid et al. [64] in another study focused on magnetic resonance imaging applications, with participants acknowledging AI’s potential to improve workflow. Giansanti et al. [65] gathered opinions on post-pandemic AI use, emphasizing the need for a structured questionnaire for scientific societies. Abuzaid et al. [66] in a third article investigated opinions on AI integration into radiology workflow, revealing low awareness and the importance of tailored training.

Alelyani et al. [67] explored attitudes toward AI among radiologists, radiographers, technologists, and students, noting varying levels of awareness and the importance of specific AI training in medical schools. The European Society of Radiology [68] extended the investigation internationally, reporting favorable positions on AI and detailed expectations for 5–10 years.

Caparros Galan et al. [69] focused on student opinions, finding a belief in AI’s potential to reform the radiology workflow without jeopardizing radiologists’ work. Di Basilio et al.’s study [70] involved professionals with diverse backgrounds and highlighted the importance of survey administration procedures.

Two surveys sponsored by different scientific societies were reported in [71,72]. Diaz et al. [71] surveyed international medical physicists, exploring training aspects and opinions on AI introduction. Coppola et al. [72] conducted a nationwide survey on Italian radiologists, examining their interactions with AI, ethical concerns, job loss risks, policy needs, and opinions.

##### Further Areas of Focus with the Point of View of the Challenges

Further domains exploring these themes as challenges encompass algorithms [73], examining the pivotal role of the responsible professional [74], delving into tools, datasets, and workflows [75], understanding the dynamics of teamwork [76], and exploring educational aspects [77]. Each of these areas merits a dedicated review [39] for a comprehensive understanding of the challenges posed by these critical components in the integration of artificial intelligence in healthcare. The challenges in algorithm development, also with a focus on the need for standardization, were explored by Fazal et al. [73]. The study highlighted macro-areas of challenges, including the need to decrease false-positive rates for computer-aided detection and to enhance the understanding of AI reasoning. Moreover, the study emphasized, from a standardization viewpoint, the importance of well-defined responsibilities in case of algorithm-induced errors. Gampala et al. [74] delved into challenges related to the professional role and responsibilities in digital radiology in the context of AI integration. The study discussed how AI could simplify various activities in the working chain, emphasizing the need for clinical teams to become familiar with guiding principles for data management and curation and to standardize it.

Ahmad [75] focused on challenges in engineering and machinery aspects, such as hardware, software, and workflow impact. The study reported on new activities, such as selecting AI products and vendors, piloting AI algorithms, and creating proprietary AI algorithms that need standardization.

Martin-Noguerol et al. [76] highlighted challenges in teamwork concerning the collaboration between engineers, system developers, and radiologists. Communication between radiologists and data scientists was deemed crucial for successful collaborative work and needs to be regulated.

Pesapane et al. faced the importance of education [77] and underscored the significance of continuous, specialized training, considering the evolving nature of AI and the need for well-defined and standardized programs.

### 4.5. Comprehensive Synoptic Overview

**Figure 6:**
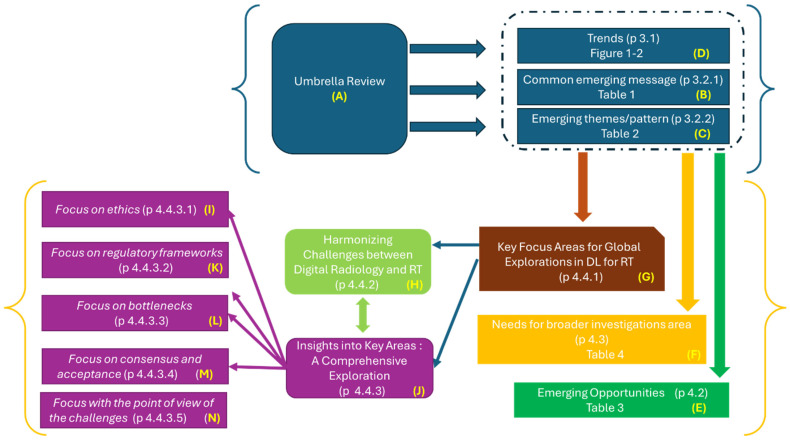
Synoptic diagram representing the flow and the key points of the study.

This extensive investigation, conducted via an umbrella review using a narrative review methodology, has allowed for the illumination of intricate themes regarding the application and integration of DL techniques within RT. This comprehensive exploration (Figure 6A) extends beyond mere thematic revelations (Figure 6B,C), encapsulating trends (Figure 6D), nuanced emerging opportunities (Figure 6E), needs for broader investigation areas (Figure 6F), and key focus areas for global explorations in DL for RT suggested from the overview (Figure 6G). The last passage suggested a further elaboration of the overview with complementary information. The complementation with a non-systematic review of recent contributions has made it possible to perform an evaluation and comparison by linking the technological challenges faced in the reality of digital radiology and AI, strictly interconnected with the investigated field of the integration of DL in RT (Figure 6H), and a comprehensive exploration of the key areas identified has been provided (Figure 6J). This exploration has facilitated a series of focused discussions on the detected key focus areas for global exploration: ethical considerations (Figure 6I), regulatory frameworks (Figure 6K), identification of bottlenecks (Figure 6L), the cultivation of consensus and acceptance within the field (Figure 6M), and the focus with the point of view of the challenges (Figure 6N).

As emerged from the synoptic diagram, the study of recent literature has allowed for a detailed exploration of the key areas identified by the systematic literature reviews initially analyzed in this umbrella review.

To summarize, the comprehensive overview, combined with the additional analytical discussion, successfully addressed the critical issues arising from the original hypothesis. However, it is important to emphasize that although a narrative review does not inherently aim for such outcomes, the in-depth nature of this investigation greatly enhances the understanding and development of the topic under discussion.

### 4.6. Takeway Message

Significant initiatives have involved the integration of DL with radiotherapy. These initiatives experienced a notable acceleration during the COVID-19 pandemic. This period highlighted significant development opportunities alongside specific niches needing improvements and further scientific exploration. Furthermore, there is a widespread call for in-depth examination regarding standardization, guidelines, access to dataset databases, and the corresponding external validations.

### 4.7. Limitations

This study utilized the PubMed and Scopus databases, focusing on systematic review studies through an umbrella review narrative. Further targeted research on emerging themes is encouraged. Exploring national databases can also enhance knowledge in this domain by identifying local initiatives, with particular emphasis on standardization and regulatory compliance.

## 5. Conclusions

In conclusion, the synthesis of the findings shed light on the profound potential of DL in revolutionizing radiotherapy. The acceleration driven in this field (as in others) by the COVID-19 pandemic has led to significant advancements that indeed have the potential to offer great opportunities in the healthcare domain. However, attention must be paid to specific areas that require further research.

The overview not only highlighted key areas poised for further exploration but also underscored the nuanced interplay between digital radiology and radiation therapy. This interdependence aids in addressing cross-cutting areas that require further developments in the realms of both research and regulation.

As technological advancements continue to progress, concerted collaborative efforts and thorough investigations are essential to fully harness the transformative power of DL. Ultimately, such endeavors hold the promise of significantly improving patient care and refining treatment strategies for optimal outcomes.

## Figures and Tables

**Table 1:** Key findings from the analysis of the overview of systematic reviews.

Review Study	Key Findings from the Analysis
Almeida et al. (2020) [21]	This study showcases DL’s potential to revolutionize prostate cancer treatment planning by automating the contouring process, leveraging CT and MRI for improved efficiency and consistency. This paper also underscores the hurdles of limited patient datasets and the need for further development before full clinical adoption is feasible.
Kothari et al. (2020) [22]	Focusing on the prognostic value of radiomics in NSCLC treated with radiotherapy, this systematic review notes heterogeneity in study methodologies and recommends standardizing radiomics features and employing robust methods and DL to improve future model performance.
Chlap et al. (2021) [23]	This study explores the critical role of data augmentation in enhancing DL models for radiology and radiotherapy, categorizing techniques for CT and MRI images to offset the need for large datasets. The study emphasizes data augmentation’s value in bolstering algorithm performance and validating clinical applicability amidst dataset limitations.
Spadea et al. (2021) [24]	This study assesses DL-based synthetic CT generation across three clinical applications, including its use in MR-based treatment planning, IGRT, and PET correction. The study examines the contributions, challenges, and future potential of DL-based sCT methods, evaluating their readiness for clinical implementation.
Huang et al. (2021) [25]	This study explores the pivotal role of computer technology and data expansion in advancing AI, offering radiation oncologists efficient tools to enhance radiotherapy and prompting a crucial need for understanding DL principles for effective clinical application. This paper also discusses AI’s potential growth in radiation oncology.
Walls et al. (2021) [26]	This study investigates radiomics’ role in lung cancer radiotherapy decisions, noting the absence of validated biomarkers for personalized treatment. Despite linking radiomic indicators to clinical outcomes, challenges, such as data standardization and validation, hinder practical application, emphasizing the need for improved research methodologies.
Kim et al. (2021) [27]	This meta-analysis evaluates the AI-assisted MRI’s diagnostic ability for distinguishing true progression from non-progression in brain metastasis post-radiotherapy, finding sensitivity and specificity rates of 77% and 74%, respectively. Despite these findings, the current diagnostic reliability of AI-assisted MRI is insufficient for clinical application.
Avanzo et al. (2021) [28]	This paper investigates AI applications in Italian imaging research from 2015 to 2020, revealing MRI as the predominant modality, notably in neurological diseases and cancer diagnosis. The study highlights a surge in AI research, particularly in classification and segmentation tasks, emphasizing the necessity for collaborative frameworks and shared databases.
Yang et al. (2022) [29]	This study analyzes the DL models for cervical cancer CT image segmentation, showing high accuracy in segmenting clinical target volumes and organs-at-risk. Despite the efficient performance, the study emphasizes the necessity for public, high-quality databases, and extensive validation for future radiotherapy applications.
Rusanov et al. (2022) [30]	This study investigates DL’s role in improving CBCT image quality for online ART, focusing on updating patient anatomy to optimize treatment parameters despite traditional CBCT limitations. This review evaluates DL strategies for CBCT correction and synthetic CT generation, concluding with recommendations for clinicians and DL practitioners.
Booth et al. (2022) [31]	This study assesses the accuracy of DL models in monitoring biomarkers for glioblastoma treatment response, revealing promising diagnostic performance in differentiating tumor progression from mimics using MRI features. Despite moderate sensitivity and specificity, the studies suffer from small sample sizes and a high bias risk, indicating a need for improved study quality to refine research methodologies.
Hasan et al. (2022) [32]	This study examines CNNs’ application in ENT radiology, revealing their high accuracy in tasks such as structure identification, pathology detection, and tumor segmentation for radiotherapy across various subspecialties. The study highlights the potential of CNN’s application to revolutionize clinical practice by automating and improving diagnostic and treatment planning processes.
Liu et al. (2023) [33]	This study assesses DL algorithms’ effectiveness in contouring organs-at-risk in HNC radiation planning, demonstrating DSC and indicating DL’s potential to automate contouring and enhance precision in radiotherapy plans. The study emphasizes the importance of quality datasets to optimize DL’s performance, potentially reducing oncologists’ workload.
Tan et al. (2023) [34]	This study explores the efficacy of DL models in predicting radiotherapy-induced toxicity across multiple cancer types, emphasizing advanced techniques, such as ensemble learning and transfer learning, and underscores the necessity for larger datasets and standardized methodologies to improve research outcomes.
Franzese et al. (2023) [35]	This study analyzes DL’s role in HNC radiotherapy, emphasizing organ-at-risk segmentation’s prominence and advocating for assessing AI’s clinical impact and confidence levels for predictions. The study concludes by highlighting AI’s potential to automate HNC radiotherapy workflows.
Eidex et al. (2023) [36]	This systematic review highlights DL’s role on MRI-guided radiation therapy in enhancing tumor segmentation, deriving X-ray attenuation from MRI, and improving tumor characterization and motion tracking, with recent trends focusing on multi-modal, visual transformer, and diffusion models.
Chen et al. (2024) [37]	This study investigates unpaired image-to-image translation in medical imaging, showcasing its applications in segmentation and clinical tasks but noting limitations, such as limited external validation and scarce pre-trained models, hindering immediate clinical application.
Boldrini et al. (2024) [38]	This systematic review explores the impact of AI, DL, and radiomics on IGRT, revealing their potential in diagnosis, treatment optimization, and outcome prediction, though further research is needed to establish their clinical impact and integration into standard protocols.

AI: artificial intelligence; ART: adaptive radiation therapy; CBCT: cone-beam computed tomography; CNN: convolutional neural network; CT: computed tomography; DSC: dice similarity coefficient; DL: deep learning; HNC: head and neck cancer; IGRT: image-guided radiation therapy; MRI: magnetic resonance imaging; NSCLC: non-small-cell lung cancer; PET: positron emission tomography.

**Table 2:** Emerging categorization from the systematic reviews analyzed.

Area of Interest	Focus on the Categorization
*Automating contouring process*	Several systematic reviews (Almeida et al. [21], Huang et al. [25], Avanzo et al. [28], Yang et al. [29], Liu et al. [33], Franzese et al. [34], and Chen et al. [37]) explore the increasing role of DL on image segmentation and automating the contouring process, permitting a reduction in the workload for physicians and enabling more precise radiotherapy plans.
*Use of radiomics*	Kothari et al. [22], Walls et al. [26], Kim et al. [27], Eidex et al. [36], and Boldrini et al. [38] discuss the increasing role of radiomics in guiding clinical decisions before, during, and after radiotherapy, highlighting the need of further research for its integration into clinical practice.
*Synthetic CT*	Spadea et al. [24] and Rusanov et al. [30] explore the innovative application of DL to generate and enhance synthetic CT (sCT) images for improved radiation therapy planning and execution. This approach addresses traditional imaging limitations by providing high-quality, accurate sCT images for a variety of clinical applications in radiotherapy.
*Application of DL for ART*	Sapdea et al. [24] and Rusanov et al. [30] investigate the use of DL to improve the quality of cone-beam CT images for guiding healthcare professionals in online ART.
*Data augmentation* *techniques for DL models*	Chlap et al. [23] and Tan et al. [34] examine data augmentation techniques for the development and improvement of DL models with various applications in radiotherapy and emphasize their necessity due to the reliance on large datasets for training.
*Use of DL for prediction of side effects and clinical* *outcome*	Several systematic reviews (Huang et al. [25], Walls et al. [26], Booth et al. [31], Tan et al. [34], Franzese et al. [35], and Boldrini et al. [38]) discuss the application and use of DL predictions of the outcome of radiotherapy and use DL to predict the toxicity and outcomes after radiotherapy.
*Improvements in treatment planning process*	Several systematic reviews (Almeida et al. [21], Hasan et al. [32], Liu et al. [33], Franzese et al. [35], Chen et al. [37], and Boldrini et al. [38]) focus on the growing role of DL techniques in radiotherapy planning optimization, highlighting the significant innovation due to introduction of DL in daily practice workflow.
*Image fusion*	Huang et al. [25] discuss the utilization of DL techniques for enhancing medical image registration across various imaging modalities (multi-time and/or multimode registration).

ART: adaptive radiation therapy; CT: computed tomography; DL: deep learning.

**Table 3:** Thematic areas within the emerging opportunities.

Opportunity	Application Area	Key Insights
*Automating Contouring in Prostate Cancer Treatment Planning*	Prostate Cancer Treatment Planning	DL automates contouring for speed and consistency, promising a paradigm shift in precision medicine [21].
*Enhanced Prognostic Precision in NSCLC*	NSCLC Radical Radiotherapy	DL-driven radiomics models refine prognostic precision, optimizing treatment strategies for NSCLC patients [22].
*Augmentation Techniques for Improved DL Performance*	Radiology and Radiotherapy Augmentation	Basic, deformable, and DL-based augmentation methods enhance DL performance, addressing limited large datasets [23].
*DL-Based Synthetic CT Generation* *Opportunities*	MR-Based Treatment Planning and Adaptive Radiotherapy	DL-based synthetic CT generation optimizes treatment parameters based on updated patient anatomy, offering precision and efficiency [24].
*Radiomics Guiding Clinical Decisions in Lung Cancer Radiotherapy*	Lung Cancer Radical Radiotherapy	Radiomics guides personalized interventions, incorporating advanced imaging features for improved clinical decisions [26].
*AI-Assisted MRI for Brain Metastasis Diagnosis*	Brain Metastasis Diagnosis	AI-assisted MRI enhances diagnostic capabilities, showing potential for influencing timely and accurate treatment decisions [27].
*AI Applications in Italian Imaging* *Research Landscape*	Imaging Research in Italy	Italy experienced a surge in AI applications, particularly in classification and segmentation tasks, emphasizing the need for collaborative frameworks [28].
*DL Precision in Cervical Cancer CT* *Image Segmentation*	Cervical Cancer CT Image Segmentation	DL models exhibit high accuracy in segmenting clinical target volumes, enhancing precision in treatment planning [29].
*Enhancing CBCT Image Quality for Online Adaptive Radiation Therapy*	Online Adaptive Radiation Therapy	Opportunities in optimizing treatment parameters based on updated patient anatomy, addressing literature gaps in CBCT image quality enhancement [30].
*Monitoring Biomarkers with DL in* *Glioblastoma Treatment Response*	Glioblastoma Treatment Response Assessment	DL offers a promising avenue for monitoring biomarkers, potentially leading to personalized therapeutic strategies [31].
*CNNs in ENT Radiology*	ENT Radiology	CNNs automate diagnostic and treatment planning processes, showcasing high accuracy within the otolaryngology community [32].
*DL Automation in Head and Neck* *Cancer Radiation Treatment Planning*	Head and Neck Cancer Radiation Treatment Planning	DL algorithms promise to automate contouring, refining precision and reducing the workload for oncologists [33].
*DL Models Predicting* *Radiotherapy-Induced Toxicity*	Radiotherapy-Induced Toxicity Prediction	DL models exhibit promise in predicting toxicity, with opportunities for refining accuracy, specificity, and applicability [34].
*DL in Complex Radiotherapy Workflow for HNC*	Complex Radiotherapy Workflow for Head and Neck Cancer (HNC)	DL’s potential in workflow automation presents opportunities for enhanced HNC care, particularly in organ-at-risk segmentation [35].
*DL in MRI-Guided Radiation Therapy*	MRI-Guided Radiation Therapy	Opportunities in enhancing tumor segmentation, deriving X-ray attenuation from MRI, and improving tumor characterization and motion tracking [36].
*Unpaired Image-to-Image Translation in Medical Imaging*	Medical Imaging	Potential game-changer in segmentation, unpaired domain adaptation, denoising, and automatic contouring, with opportunities for exploration [37].
*Integration of AI, DL, and Radiomics in IGRT*	Image-Guided Radiation Therapy (IGRT)	Significant influence on IGRT across all workflow phases, with opportunities for further exploration in diagnosis, treatment optimization, and outcome prediction [38].

**Table 4:** Thematic areas and key suggestions for a broader investigation for each reference.

Thematic Area	Key Suggestions for Broader Investigation	References
*Integration of AI and Radiomics* *in IGRT*	Conduct further studies to confirm the impact of AI and radiomics on IGRT, emphasizing the need for evidence beyond retrospective data.	[38]
*Advanced Contouring Technologies*	Focus on constructing high-quality datasets for automated contouring technology using DL algorithms in head and neck OARs. Enhance DL performance through algorithm optimization and innovation.	[33]
*Toxicity Prediction in Radiotherapy*	Utilize large and diverse datasets for toxicity prediction, emphasizing the standardization of study methodologies to improve the consistency of research outcomes.	[34]
*Automation of HNC Treatment Workflow*	Align the development of AI technologies in HNC treatment with clinical needs by conducting interdisciplinary studies involving clinicians and computer scientists.	[35]
*Application of CNN Methodology in ENT Radiology*	Explore the potential uses of CNN methodology in ENT radiology, including nodule and tumor identification, anatomical variation identification, and tumor segmentation. Encourage continued evolution of technologies in everyday practice.	[32]
*Image-to-Image Models and* *External Validation*	Address the scarcity of external validation studies for image-to-image (I2I) models, emphasizing the need for publicly available pre-trained models to enhance the immediate applicability of proposed methods.	[37]
*Automatic Segmentation in Cervical Cancer*	Despite good accuracy in automatic segmentation of CT images for cervical cancer, future investigations should focus on obtaining public high-quality databases and conducting large-scale research verification.	[29]
*Recommendations for Clinical* *Practice*	Provide recommendations for clinicians and DL practitioners based on literature trends and the current state-of-the-art DL methods in radiation oncology.	[30]
*Diagnostic Performance Using MRI Features*	Recognize the good diagnostic performance of ML models using MRI features but suggest improvements in study quality and design for enhanced reliability.	[31]
*DL-Based Synthetic CT Generation*	Evaluate the clinical readiness of DL-based synthetic CT generation methods and suggest further initiatives for their potential implementation.	[24]
*Techniques Using Augmented Data*	Explore techniques using augmented data in clinical settings and build confidence in the validity of models produced.	[23]
*Future Studies for AI-Assisted MRI*	Acknowledge the need for future studies with improved methodologies and larger training sets to enhance the diagnostic performance of AI-assisted MRI in clinical practice.	[27]
*AI Applied to Imaging in Italy*	Recognize the unprecedented interest in AI applied to imaging in Italy and suggest initiatives for building common frameworks, databases, collaborations, and guidelines for research on AI.	[28]
*Standardized Radiomics Features and Deep Learning*	Advocate for future research focusing on standardized radiomics features, robust feature selection, and deep learning techniques to improve prognostic capabilities in lung cancer models.	[22]
*Development of AI in Radiation* *Oncology*	Explore the development and basic concepts of AI in radiation oncology based on different task categories of DL algorithms. Clarify the potential for further DL development in the field.	[25]
*Continuous Improvement of Models*	Acknowledge the satisfactory results achieved by models but highlight the need for continuous improvement before safe and effective clinical practice.	[21,22,23,24,25,26,27,28,29,30,31,32,33,34,35,36,37,38]
